# *Pasteurella multocida* Septic Shock: Case Report and Literature Review

**DOI:** 10.1155/2019/1964161

**Published:** 2019-11-03

**Authors:** A. Aljameely, G. Wali

**Affiliations:** ^1^Department of Internal Medicine, King Faisal Specialist Hospital and Research Center, Jeddah, Saudi Arabia; ^2^Head Section of Infectious Diseases, Department of Internal Medicine, King Faisal Specialist Hospital and Research Center, Jeddah, Saudi Arabia

## Abstract

*Pasteurella multocida* is a small, Gram-negative, facultatively anaerobic coccobacillus that inhabits the normal microbiota of the respiratory tract of several animals, especially cats and dogs. By infecting humans, a wide range of clinical pictures can evolve varying from mild local cellulitis to more severe systemic diseases (e.g., meningitis, pneumonia, endocarditis, and bacteremia). Septic shock is an uncommon complication of *P. multocida* infection, with less than 100 cases reported in the literature. It is frequently associated with cirrhotic and immunocompromised individuals and rarely immunocompetent ones. Here, we present a case of *Pasteurella multocida* septic shock in an elderly man secondary to leg cellulitis with a review of the relevant literature.

## 1. Introduction

*Pasteurella multocida* is a small, Gram-negative, facultatively anaerobic coccobacillus that inhabits the normal microbiota of the respiratory tract of several animals, especially cats and dogs. By infecting humans, a wide range of clinical pictures can evolve varying from mild local cellulitis to more severe systemic diseases (e.g., meningitis, pneumonia, endocarditis, and bacteremia) [1]. Septic shock is an uncommon complication of *P. multocida* infection, with less than 100 cases reported in the literature [2]. It is frequently associated with cirrhotic and immunocompromised individuals and rarely immunocompetent ones [3]. Here, we present a case of *Pasteurella multocida* septic shock in an elderly man secondary to leg cellulitis with a review of the relevant literature.

## 2. Case Report

A 75-year-old male presented to our emergency department with a one-day history of productive cough and shortness of breath preceded by five days history of left leg redness, hotness, and pain. He sought medical care in the early days of symptoms development and prescribed clindamycin as treatment of mild skin and soft tissue infection. Nevertheless, an improvement was not reported.

On systematic review, there was no fever. No chest pain, palpitation, or hemoptysis. No gastrointestinal or genitourinary symptoms.

There was no history of leg trauma or penetrating injury. No history of recent travel or IV drug use.

His medical background is significant for multiple comorbidities including type II diabetes mellitus, hypertension, dyslipidemia, hypothyroidism, heart failure, stage II chronic kidney disease, obstructive sleep apnea, and morbid obesity. Of note, he had several previous documented episodes of mild lower limb cellulitis. No past surgical history. He is an ex-smoker with a history of more than 30 pack-years. The patient reported no history of alcohol abuse.

On examination, he was conscious, oriented, distressed, and ill-looking. Vital signs were unstable, and as follows: BP 83/38 mmHg, RR 24, O_2_ sat 88% on RA, HR 100 bpm, and temperature 37.8°C.

**Figure 1:**
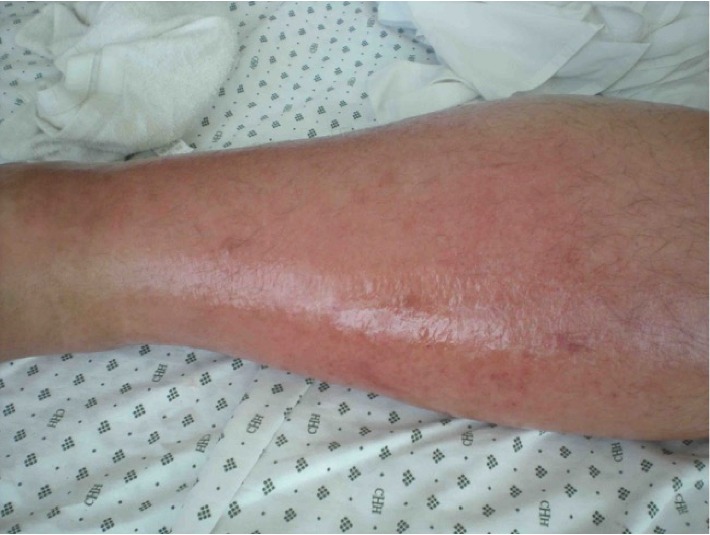


Systems examination was nonrevealing. Lower limb assessment showed a mildly tender left leg with diffuse, ill-demarcated erythema involving the anterior compartment up to the knee with no significant swelling. Crepitus was not appreciated, and peripheral pulse was palpable (Figure 1).

Initial laboratory parameters were showing marked leukocytosis (WBC: 21 × 10^9^/L) with neutrophilic shift (absolute neutrophilic count: 15.8 × 10^9^/L), elevated levels of lactic acid (lactic acid: 4.8 mmol/L), and acute kidney injury picture (Cr: 182 *μ*mol/L–60 mmol above his baseline of 120 *μ*mol/L). CXR demonstrated an obliterated left costophrenic angle with new infiltrates over his right lower lobe.

In light of the above-mentioned observations, the patient was admitted to a critical care area where resuscitation was immediately started. Pressors were initiated in the form of dopamine, and optimal oxygenation was provided by a 3–5 L O_2_ face mask.

A septic screen was sent, and he was started on IV vancomycin and levofloxacin.

The second day, his vital parameters normalized and pressors weaned off. Blood culture was finalized after 72 hours using the VITEK®-2 microbial identification system as *Pasteurella multocida* (Table 1). Hence, vancomycin was discontinued.

At this point in time, the patient was further questioned about animal contact, scratches, or bites.

He reported that he had played with his daughter's cat, which scratched his legs multiple times in the last year, last one almost a week prior to his presentation.

On the fifth day, the patient had an allergic reaction to levofloxacin in the form of generalized urticarial skin rash, so it was stopped and amoxicillin-clavulanic acid started. Repeated blood culture confirmed clearance. The patient's clinical status and laboratory parameters were followed during his 6 days of hospitalization, which showed excellent response to treatment. He was discharged home in a stable condition on amoxicillin-clavulanic acid 625 mg oral tablets three times per day to complete a total of 14 days of antibiotic therapy. Two weeks follow-up in the OPD setting showed complete resolution of symptoms.

## 3. Discussion

Among the *Pasteurella* genus, five species are usually implicated in human-related infections: *P. multocida*, *P. septica*, *P. canis*, *P. stomatis*, and *P. dagmatis* [4], out of which, *P. multocida* is the commonest [5]. It resides in the normal microbiota of many animals' nasopharynx and respiratory tracts, importantly cats and dogs, and transmitted to humans through several ways including licking, scratching, and most frequently biting [5, 6]. Pasteurellosis has a wide spectrum of clinical syndromes, and as described in our case, skin and soft tissue infection followed by respiratory infections are by far the most common forms [4, 7]. Septic shock is a severe and rare complication of *Pasteurella* infection [2]. Multiple risk factors have been associated with increased risk for developing septicemia secondary to pasteurellosis, and all of them imply a disturbed and compromised immune system and these notably include advanced age, chronic liver disease, and diabetes mellitus [8]. However, pasteurellosis in otherwise healthy individuals has been reported with much lower frequency [8]. Liver dysfunction has been established by multiple previous reports as a major risk factor for pasteurellosis, especially septicemia [5, 7]. Chronic obstructive pulmonary disease is reported as an associated condition with pasteurellosis of the respiratory tract [4, 8]. In contrast to peritoneal dialysis, where *P. multocida*-associated peritonitis is reported multiple times in the literature, septic shock secondary to pasteurellosis in hemodialysis patients was reported only two times [9, 10]. Animal exposure is almost always present in cases of pasteurellosis; however, its absence does not rule out the disease. In one study, 47 patients with *Pasteurella* septicemia were reviewed and cat and dog exposure was reported in 26 and 10 patients, respectively, where 8 patients had no exposure and the remaining 3 reported different animal exposures [8].

Penicillin, ampicillin, and amoxicillin are the proper antibiotic regimen for *P. multocida* infections [1, 6]. Doxycycline and fluoroquinolones can be used in penicillin-allergic patients [1, 6]. In our case, and in accordance with the literature, levofloxacin and amoxicillin were quite effective. Alternative antibiotic choices include second- and third-generation cephalosporins and chloramphenicol [1]. First-generation cephalosporins, erythromycin, antistaphylococcal penicillins, aminoglycosides, vancomycin, and clindamycin are not effective against *P. multocida* and have poor activity in vitro [1].

## 4. Conclusion

Given the possible associated mortality with *Pasteurella multocida* septicemia, detailed history of animal exposure, especially cats and dogs, must be carried out in the management of a patient presenting with septic shock and possible skin and soft tissue infection. Presence of risk factors such as immunocompromised status raises the suspicion index about a possible role of *Pasteurella multocida*; however, their absence does not rule it out.

## Figures and Tables

**Table 1:** Blood culture and sensitivity panel.

-	*Pasteurella multocida*	MIC	Interpretation
1	Benzylpenicillin	0.125	S
2	Amoxicillin/clavulanate	0.25	S
3	Ceftriaxone	0.016	S
4	Levofloxacin	0.032	S
5	Erythromycin	8	R
6	Trimethoprim/sulfamethoxazole	0.25	S

MIC: minimum inhibitory concentration; S: sensitive; and R: resistant.
